# Titanium elastic nailing can be used in 6 to 10 years old pediatric with Delbet IV femoral neck fractures

**DOI:** 10.1097/MD.0000000000027588

**Published:** 2021-10-29

**Authors:** Dongsheng Zhu, Xiangfei Xu, Ming Zhang, Tong Wang

**Affiliations:** Department of Pediatric Surgery, The First People's Hospital of Lianyungang, Affiliated to Xuzhou Medical University, Lianyungang, China.

**Keywords:** femoral neck fracture, pediatric, titanium elastic nail

## Abstract

The purpose of this study was to analyze the outcomes of titanium elastic nail (TEN) for the children in 6 to 10 years old who sustained a Delbet IV femoral neck fracture.

A total of 56 children aged 6 to 10 years old with Delbet IV femoral neck fracture treated with TEN or cannulated screw (SC) were identified at our hospital from January 2009 to December 2019. Of which 24 were treated with TEN, and 32 with SC. All of them were followed up for 1 year after operation, and the differences in operation time, intraoperative blood loss, hospitalization time, hip joint function, and complication between the 2 groups were compared. Harris and Ratliff hip score were used to evaluate the hip function.

All 56 fractures united properly. No major complications were noted in both groups. The intraoperative blood loss and operation time in TEN group and SC group were (11.42 ± 3.41) mL, (19.66 ± 4.05) mL (*P* = .000) and (33.58 ± 7.89) min, (40.22 ± 7.48) min (*P* = .002), respectively. There was no significant statistical difference between hip regarding range of motion and femoral neck-shaft angle in both groups, as well as Harris and Ratliff hip score between the 2 groups.

TEN represent safe and effective methods in the treatment of Delbet IV femoral neck fracture in 6 to 10 years old children. TEN internal fixation is a minimal invasive and simpler technique and suitable for young children of Delbet IV femoral neck fracture.

## 1 Introduction

Due to covering of thick and strong periosteum, as well as tough strong bone, fractures of the femur are uncommon injuries in children.^[1,2]^ Pediatric femur neck fracture is exceedingly rare and accounts for no more than 1% of all pediatric fractures according to reports.^[3–5]^ Despite their rarity, these fractures are associated with high rates of coxa vara, delayed union, and nonunion, especially osteonecrosis in the treatment without internal fixation.^[6,7]^ And premature epiphyseal closure, limb length discrepancy, and osteonecrosis also occur and are more common with operative treatments.^[8]^ Kirschner wire and cannulated screw (SC) are the preferred treatment in adolescents, however, the effective time of Kirschner wire is only 6 weeks, so SC is more suitable for school-age children, because they owning a longer healing time.^[9]^ Regrettably, SC internal fixation will destroy the periosteum of femur, and it will increase the risk of osteonecrosis of the femoral head in theory. Several recent studies suggested that titanium elastic nail (TEN) not damage the periosteum, and become the ideal device for care for most pediatric long bone.^[10]^ However, no studies focused on the treatment by TEN of the children suffering from femur neck fracture. The purposes of this study are to compare outcome of TEN and SC in Delbet IV femur neck fracture in 6 to 10-years children and to share our experience in the treatment of Delbet IV femur neck fracture.

## 2 Patients and methods

### 2.1 Patients

A retrospective analysis of the femur neck fractures database at The First People's Hospital of Lianyungang (Lianyungang, China) was performed, after obtaining approval from the respective Institutional Review Board. In total, 56 patients were included; each patient underwent surgery for femur neck fractures were diagnosed by X-ray between January 2009 and December 2019. The patients were recruited for the current study if they were 6 to 10 years old with Delbet IV femur neck fracture, and were willing to participate in this study and sign the written informed consent. The exclusion criteria were as follows: pathologic fractures; pathologic fractures; and fractures in patients suffering from neuromuscular disorders. All patients provided written informed consent, and this study was conducted in accordance with the Declaration of Helsinki.

### 2.2 Surgical procedures

TEN, as a disposable, single-use device (Trauson Medical Instrument Technology Co., Ltd., Changzhou City, Jiangsu Province, China), was employed for operation of children in TEN group. Whereas, a 1-time SC (Wego Medical Instrument Technology Co., Ltd., Weihai City, Shandong Province, China) was applied for the individuals in the SC group. The operative methods of the 2 groups (TEN group^[11]^ and SC group, respectively) were in accordance with previous literature.^[9]^ Surgery was performed with the patient in the supine position on a traction table. The nail size is chosen in relation to the diameter of the medullary canal (2.5–4.0 mm) with a nail size/medullary canal diameter ratio of 40%. The operation was performed by the same team of doctors.

### 2.3 Evaluation of operation

In order to evaluate clinical outcomes, we measured and recorded various intraoperative and postoperative parameters, including age, weight, sex, operative time, blood loss during operation, and length of hospitalization in 2 aforementioned groups. The operation time was recorded from the initiation of the surgery to the end of surgery. Intraoperative blood loss was measured using a piece of completely soaked gauze (5 cm × 5 cm), which represented an average carrying capacity of 5 mL blood.^[12]^

### 2.4 Follow-up observation

Postoperative follow-up was conducted based on the analysis of X-ray images after surgery. After operation, spica casting with hip and knee for 4 weeks was applied for all patients. Weight bearing was not allowed within 2 months. Anteroposterior and lateral digital radiographs of the entire affected femur were obtained postoperatively at 4, 8, and 12 weeks. The radiographic data included full-length anteroposterior femur, lateral views of femur, and view of pelvis. Hip ranges of motion and femoral neck-shaft angle were also measured. The final functional outcomes evaluated by using Harris scoring system^[13]^ and Ratliff clinical and radiographic assessment system.^[14]^

### 2.5 Identification of sample size

Based on our pilot data, the sample size was estimated at a study power of 80% and a significance level of 5% using the PASS11 software (NCSS, Kaysville, Utah). It was suggested that at least 22 patients were required per group.

### 2.6 Statistical analysis

Statistical analysis was performed using SPSS 20.0 statistical software (SPSS Inc., Chicago, IL) by one of the authors. Measurement data were presented as means ± standard deviation (range: minimum–maximum). The numerical variables were analyzed using the *t* test. Meanwhile, the categorical data were expressed as numbers, which were analyzed by Pearson χ2 and Fisher exact text. Two-sided *P* value <.05 was considered as statistical significance.

## 3 Results

A total of 56 patients (26 boys and 30 girls) met the inclusion criteria. Twenty-four patients were treated with TEN method and 32 patients were treated with SC method. The average age of the patients was 7.92 ± 1.50 years (range, 6–10 years), 7.97 ± 1.49 years (range, 6–10 years) and the average weight was 28.04 ± 7.64 kg (range, 18–45 kg), 29.69 ± 8.21 years (range, 18–48 years), respectively. The TEN group was hospitalized on an average of 6.46 days, while the SC group was hospitalized on an average of 6.44 days. There were 11 cases of right femur neck fracture and 13 cases of left femur facture in TEN group. And the number of which was 16 and 16 in SC group. We noted no statistically significant difference between the 2 groups in terms of all above characteristics. However, there was a significant difference in time of operation, and bleeding during the operation (Table 1).

**Table 1:** Patient characteristic.

Variable	TEN group (N = 24)	SC group (N = 32)	χ^2^/*t* value	*P* value
Age, yr			0.129	.898
Range	6 to 10	6 to 10		
Median	7.92 ± 1.50	7.97 ± 1.49		
Weight, kg			0.765	.448
Range	18 to 45	18 to 48		
Median	28.04 ± 7.64	29.69 ± 8.21		
Sex, n			0.383	.536
Male	10	16		
Female	14	16		
Laterality, n			0.095	.757
Left	13	16		
Right	11	16		
Operation time, min			3.210	.002
Range	19 to 52	28 to 56		
Median	33.58 ± 7.89	40.22 ± 7.48		
Blood loss, mL			8.044	.000
Range	8 to 20	10 to 26		
Median	11.42 ± 3.41	19.66 ± 4.05		
Length of hospitalization, d			0.100	.920
Range	5 to 8	5 to 8		
Median	6.46 ± 0.72	6.44 ± 0.80		

**Figure 1:**
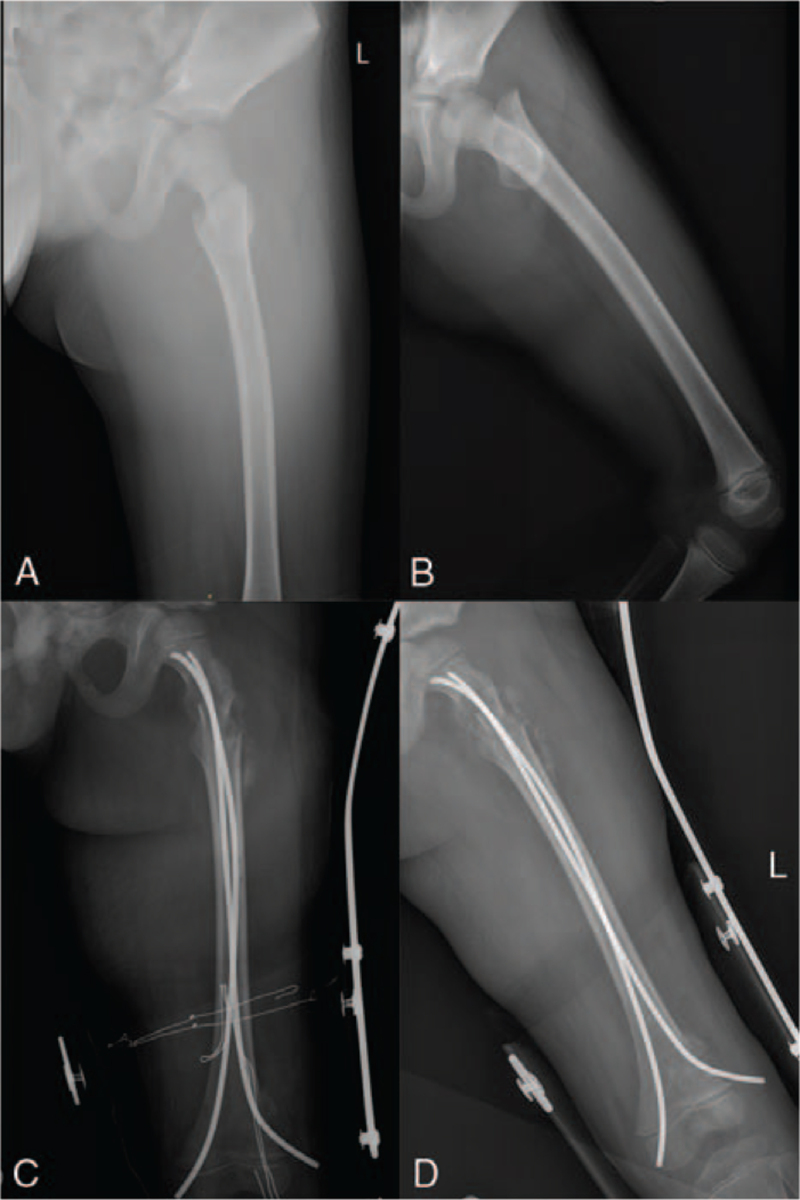
An eight-year-old male fell down from a height of 1 m. A and B: Before operation. C and D: After operation.

At the final follow-up, all patients achieved good stability of walking and were able to walk without limping. The range of motion in the hip joint with no difference between the 2 groups (*P* > .05) (Table 2). According to the Ratliff clinical and radiographic assessment system (Table 3), good and fair results were demonstrated in 92% and 8% of patients, respectively in the TEN group and in 84% and 16% of patients, respectively in the SC group. No poor results were observed in both groups, there was a significant difference (Table 4). Harris outcome scores given similar results (Table 5). During follow-up, TEN group recovered well (Fig. 1).

**Table 2:** Comparison of hips ROM and NSA after operation.

Variable		TEN group	SC group	*t* value	*P* value
ROM of hip, °	Flexion	134.25 ± 2.92	135.22 ± 2.96	1.219	.228
	Extension	12.75 ± 1.62	12.31 ± 1.82	0.931	.356
	Abduction	37.71 ± 4.48	36.97 ± 4.15	0.638	.526
	Adduction	25.79 ± 2.89	25.06 ± 3.04	0.908	.368
	Extorsion	34.83 ± 2.93	34.50 ± 3.02	0.414	.680
	Intorsion	44.92 ± 3.26	44.66 ± 3.18	0.300	.765
NSA, °	129.71 ± 3.32	127.84 ± 4.67	1.665	0.102	

**Table 3:** Ratliff clinical and radiographic assessment system.

Good	Clinically, no or negligible pain, full or minimal restrictive hip movement, and normal activity or the avoidance of games. Normal or some deformity of the femoral neck in the radiograph
Fair	Clinically, occasional pain, hip movement restriction less than 50%, and normal activity or the avoidance of games. Severe deformity of the femoral neck, mild avascular necrosis in the radiograph
Poor	Clinically, disabling pain, hip movement restriction more than 50%, and restricted activity. Severe AVN, degenerative arthritis, arthrodesis in the radiograph

**Table 4:** Ratliff outcome scores.

outcome	TEN group	SC group	χ^2^ value	*P* value
Good, n (%)	22 (92)	27 (84)	0.667	.414
Fair, n (%)	2 (8)	5 (16)		
Poor, n (%)	0 (0)	0 (0)		

**Table 5:** Harris outcome scores.

outcome	TEN group	SC group	χ^2^ value	*P* value
Excellent, n (%)	22 (92)	27 (84)	0.715	.699
Good, n (%)	1 (4)	2 (6)		
Fair, n (%)	1 (4)	3 (10)		
Poor, n (%)	0 (0)	0 (0)		

The complication rates in both groups were low and similar (16% vs 15%, *P* = .916) (Table 6). Complications were classified into major and minor. Major complications include nonunion, coxa vara, avascular necrosis (AVN), premature epiphyseal closure, and refracture. Major complication may require unplanned surgical intervention; otherwise it will affect growth and/or function. Minor complications include irritation, infection, bleeding, malalignment, leg length inequality, and delayed union. The most common complication in the TEN group was pain from prominent nail end (2 patients), 1 patient with 6° to 10 ° of fracture malalignment at the time of radiographic union was found and the malalignment had remodeled at the time of final follow-up. One patient, who was treated with TEN, demonstrated leg-length inequality where the affected limb was 1.0 cm longer. One patient in SC group was found with 1 cm shorter leg. One AVN and 1 coxa vara were found in SC group. No patient underwent unplanned surgery for complications in both groups.

**Table 6:** Complications of 2 types of surgeon in children.

Variables	TEN group	SC group	χ^2^ value	*P* value
Infection, n (%)	0 (0)	1 (3)		
Bleeding, n (%)	0 (0)	1 (3)		
Irritation, n (%)	2 (8)	0 (0)		
Malalignment, n (%)	1 (4)	0 (0)		
Leg length inequality, n (%)	1 (4)	1 (3)		
Refracture, n (%)	0 (0)	0 (0)		
Premature epiphyseal closure, n (%)	0 (0)	0 (0)		
Nonunion, n (%)	0 (0)	0 (0)		
Coxa vara, n (%)	0 (0)	1 (3)		
Avascular necrosis, n (%)	0 (0)	1 (3)		
Total, n (%)	4 (16)	5 (15)	0.011	.916

## 4 Discussion

According to literatures, fractures of the femoral neck are uncommon in the pediatric population.^[14,15]^ Because the femoral neck of children is dense and hard compared to adult femoral neck, femoral neck fractures in children are always a result of high energy trauma.^[16]^ Femur neck fractures were classified to 4-part classification system: type I is a traumatic disruption of the proximal femoral epiphysis, type II is an intra-articular transcervical fracture, type III is an intra-articular cervicotrochanteric fracture, and type IV is an extracapsular intertrochanteric fracture.^[17]^ It was initially described by Delbet and popularized by Colonna. Most of the studies on femoral neck fractures in children reported Delbet type II fractures as the most common, followed by type III and type IV. Type I fractures (transepiphyseal) are extremely rare in the literature.^[2,14]^

It can be seen that femoral neck fractures in children are rare; however, the complications are extremely serious. Many studies have shown that the incidence of complications varied between 20% and 50%, also have pointed out that nonoperative treatment of femoral neck fractures in children has poor results.^[18,19]^ AVN is the leading cause of poor prognosis as little can be done to salvage the affected hip, especially in some older literature the high rate of AVN was present 45% reported by Ratliff,^[15]^ even 58% by Hamilton.^[20]^ In contrast, with the adoption of surgical treatment, Azouz et al^[21]^ reported a very low incidence of AVN, only 13%. Therefore, early surgical treatment of femoral neck fractures in children is recommended.^[8]^

At present, the traditional surgical methods for femoral neck fractures include Kirschner wires, SC and even hip locking compression plate, among which SC internal fixation has gradually become the mainstream.^[9]^ Simultaneously, there were some reports on the experience of TEN in the treatment of subtrochanteric fractures of the femoral neck in children.^[22]^ But no TEN have been reported in the treatment of femoral neck fracture. In this research, we retrospectively compared TEN with SC for the treatment of Delbet IV femoral neck fractures in 6 to 10 years children. The results showed that both TEN and SC method can obtain good functional outcomes according to Ratliff score or Harris score. The rate of “excellent” and “good” results was similar in both groups. We also found TEN had a similar complication rate when compared with SC (16% in TEN group vs 15% in SC group). Currently, the diameter of SC is generally 4.0 mm or 4.5 mm, for older children can even reach upto 7.7 mm, however, the diameter of TEN is only 2.5–4.0 mm, in most of children 2.5 mm are most used. Furthermore, SC fixation needs to penetrate the epiphysis and reach the femoral head, which will damage the epiphyseal plate and affect the blood supply of the femoral head. And the larger the diameter, the greater the potential damage will has. As a new material and technology, children's TEN was first successfully applied by Metaizeau et al^[23]^ in Nancy, France, in 1980. Due to the curative treatment, mild trauma and simple removal, many scholars have suggested that TEN are an ideal technique for the treatment of fractures in children in recent years.^[10,11,24]^ For older or heavier children, because the biomechanical properties of TEN cannot be fully utilized, must carefully when choosing treatment. Moroz et al^[25]^ noted that children older than 11 years and heavier than 49 kg are more likely to have a complication or a poor outcome. They found that the odds ratio for poor outcome was 3.86 for children aged 11 years and older compared with those who were below this age, and children weighing more than 49 kg were 5 times more likely to have a poor outcome than those weighing less than this. In pediatric tibia and femur fractures, Andreacchio et al^[26]^ found it had potential risk factors associated with poor outcomes in children and adolescents heavier than 50 kg. In femoral shaft fractures, there were also similar findings.^[27]^ Based on our results, In terms of operation time and bleeding, TEN group was even better than SC group. Therefore, in our opinion, TEN is suitable for Delbet IV femoral neck fractures in 6 to10 years children.

The advantages of TEN in the treatment of fractures in children: it would exploit a child's denser bone, rapid healing, and ability to remodel, without risking the epiphysis or blood supply to the femoral head^[28]^; it allows a biological environment that enhances both the rate of fracture healing and the quantity of callus formation^[29]^; the elasticity of the construct allows for the ideal of micro-motion for rapid fracture healing^[30]^; provides biomechanical stability such as axial, lateral, bending resistance, and rotation resistance^[10]^; It only has two minimally invasive incisions at the metaphysis , that avoid damage to epiphyseal plate and do not affect blood supply; and the incision is beautiful in appearance and the internal fixation is easy to remove. Based on the advantages above, we think TEN has great superiority in treating of Delbet IV femoral neck fractures in 6 to 10 years children.

And the complication here is in agreement with other literature reported: irritation. To avoid soft tissue irritation, only a small amount of nail is left outside the distal metaphyseal cortex, and the nail should not be bent out into the soft tissues.

Nonetheless, the limited sample size and data collection from a single-center population may lead to deficiencies of the current study. Although our experience have above limitations, we believe our findings shed light and provide clinical values on the treatment of children femoral neck fractures.

## 5 Conclusion

In summary, our observations and discoveries suggest that TEN surgical methods have their own advantages and drawbacks. Strikingly, the application of TEN is recommended for the treatment of Delbet IV femoral neck fracture in 6 to 10 years old children. TEN represents a safe, effective, minimal invasive, simple, and suitable method.

## Author contributions

DSZ and TW conceived and directed the project. DSZ contributed to the writing of the manuscript. DSZ analyzed the data. DSZ, XFX, and MZ collected the clinical data. All authors read and approved the final manuscript.

**Conceptualization:** Dongsheng Zhu, Xiangfei Xu, Ming Zhang, Tong Wang.

**Data curation:** Dongsheng Zhu.

**Formal analysis:** Dongsheng Zhu.

**Investigation:** Dongsheng Zhu.

**Methodology:** Dongsheng Zhu.

**Project administration:** Dongsheng Zhu.

**Resources:** Dongsheng Zhu.

**Software:** Dongsheng Zhu.

**Supervision:** Dongsheng Zhu.

**Validation:** Dongsheng Zhu.

**Visualization:** Dongsheng Zhu.

**Writing – review & editing:** Dongsheng Zhu.
